# Adoption of food safety measures in smallholder dairy farms in Kenya: Implications for milk safety and public health

**DOI:** 10.1016/j.onehlt.2026.101342

**Published:** 2026-01-29

**Authors:** Ndungu Nyokabi, Emmanuel Muunda, Henrietta Moore, Luke Korir, Asaah Ndambi, Charles Omanga, Lilian Korir, Lisette Phelan

**Affiliations:** a Institute for Global Prosperity, University College London, 149 Tottenham Court Road, W1T 7NE London, United Kingdom; b University of Edinburgh Business School, 29 Buccleuch Place, Edinburgh, EH8 9JS Edinburgh, Scotland, United Kingdom; c International Livestock Research Institute (ILRI), P.O. Box 30709, Nairobi 00100, Kenya; d Faculty of Veterinary Medicine, University of Calgary, CWPH Building, 3280, Calgary, AB T2N 4Z6, Canada; e Wageningen Livestock Research, Wageningen University and Research, De Elst 1, 6708 WD Wageningen, the Netherlands; f Directorate of Veterinary Services, Private Bag, 00625 Kangemi*,* Kenya; g Lincoln Institute for Agri-Food Technology, University of Lincoln, Riseholme Park Lincoln, LN2 2LG, United Kingdom; h School of Sustainable Food and Farming, Harper Adams University, United Kingdom

**Keywords:** Milk quality, Technology adoption, Food security, Animal health, Good agricultural practices (GAPS), Milk hygiene, Smallholder dairy systems

## Abstract

Demand for animal-source foods (ASF) is growing globally, and the consumption can improve food and nutrition security. However, there are growing food safety risks associated with milk contamination. Studies assessing food safety measures (FSM) at the farm level are still limited. This study investigated FSM adoption in Kenyan smallholder dairy farms. Data were collected through focus group discussions (FGDs) and a farm survey involving 652 farmers, which considered 11 milking hygiene, 6 milk storage, 6 environmental hygiene, and 7 animal health measures.

The Food Safety Index (FSI) was 51.67%, which reveals average FSM adoption and a good agricultural practices (GAPs) compliance gap that exposes consumers to public health risks. FSM adoption was associated with participation in formal and/or informal milk value chains, socio-economic and demographic factors, knowledge of milk quality standards and regulations, farm biophysical conditions, market dynamics and institutional factors.

There is a need for increasing FSM adoption through improving farmers' knowledge, supporting their access to financial resources, and providing infrastructure and services, including roads, inputs, extension and animal health services. Policymakers should design and implement policies that address specific farmers' needs. There is also a need to provide economic incentives and ensure that the market rewards dairy producers who adopt high levels of FSM that lead to safer milk. Finally, promoting the One Health approach can help farmers address human, environmental and animal health risks, which can reduce food safety risks in dairy value chains.

## Highlights

• Food safety measures (FSM) are important in ensuring food safety.
• Results show adoption gap in smallholder dairy farms in Kenya.
• Low adoption of FSM at the farm level which poses human, animal and environmental health risks.
• Understanding the drivers of FSM adoption can provide leverage points to increase adoption.
• There is an imperative for policymakers to invest in improving farmers' knowledge of FSM.

## 1 Introduction

Consumption of animal-source foods (ASF) has the potential to improve food and nutrition security in emerging economies of the global south [1]. Milk is an important ASF that is highly nutritious, and an important source of macro and micronutrients, and constitutes an important part of diets worldwide [2]. Globally, the dairy sector is growing rapidly, driven by urbanisation, rising incomes and changing lifestyles, leading to a higher per capita consumption [1], [3]. The growth of the dairy sector in emerging economies of the global south, in particular, provides opportunities for the intensification of smallholder dairy production to contribute to food security and drive pro-poor economic development [3].

Milk production is a crucial source of income and livelihood for smallholder dairy farmers [1]. The majority of consumers in low-income countries buy milk and dairy products in informal markets made up of small-scale, unregulated market actors who dominate the milk trade [4], [5]. There are increasing calls for food quality and safety to be improved due to public health risks driven by reports of food scares and scandals publicised by the media [3]. There is also a growing body of literature showing that consumers are willing to pay for improved food safety [2], [3], [6]. Food is a known conduit for pathogen transmission, and milk is a risky food product from a public health point of view [3], [7]. Compliance with food safety standards to mitigate food-borne diseases continues to be a major challenge in the emerging economies of the Global South [1].

Milk quality is regulated by public and private standards developed to ensure and assure consumers of food safety [6]. In efforts aimed at ensuring food safety and quality compliance, low-income countries have focused on exporting products, with less emphasis placed on domestic market products [1], [6]. Poor compliance with standards affects the incomes, employment opportunities and welfare of farmers and consumers [6]. Improving food safety, an integral component of food security, is thus an important step in addressing food security challenges in low-income countries [2], [3].

The majority of the studies in emerging economies of the global south have, however, focused on value chains and explored the post-farm-gate drivers of milk quality [4], [8]. These studies show the contribution of unhygienic milk handling practices to poor milk quality in these value chains. Evidence shows that the use of plastic containers for both milking and milk storage contributes to poor milk quality [1]. The presence of contaminants in milk has been attributed to the contaminated environment in which cows are housed and/or milked, and/or deliberate adulteration with preservatives [1]. However, in the context of Eastern Africa, literature is still lacking on the assessment of the current status of the food safety measures (FSM) at the farm level. FSM are any safety practices implemented at the farm level aimed at preventing physical, chemical and microbial milk contamination [9]. These FSM can be grouped into milking hygiene, milk storage, animal health and environmental hygiene measures [6], [7], [10], [11].

Milk quality challenges are embedded across all levels and stages of milk value chains [1], [4]. However, there is a growing focus on the production stage (the first stage of the value chain) due to the high probability of milk contamination at the farm level [10], and because once milk is contaminated at the farm level, the contaminant is carried through and sometimes multiplies along the chain [12], [13]. High-quality milk facilitates marketing and is a necessity to comply with food safety standards [3]. There have been only a handful of studies that look at farm-level FSM compliance in emerging economies of the global south, mainly in India and Nepal [6], [7], [10], [11], Ethiopia [14], [15], [16], Tanzania [1] and Kenya [9]. FSM adoption at the farm level is crucial for improved milk quality [11].

Food safety issues are complex in nature and require an integrated approach that guarantees milk quality from farm to glass [10]. Several approaches employ similar principles to one health to address food safety, including literature on biosecurity and FSM at the farm level and value chain level [17], [18], [19]. Currently, there is a paucity of literature on the on-farm adoption of FSM using a holistic systems approach, such as the on-farm implementation of the One Health approach to address food safety by considering the risks that occur at the human-animal-environmental interface. The One Health approach provides an opportunity to address food safety risks related to milking hygiene, milk storage, environmental hygiene and animal health measures, including the different hazards (i.e. biological, chemical, or physical) that may enter milk either accidentally or intentionally [18], [20].

This study focuses on the Kenyan dairy sector due to its importance to food security and the role it plays in the economy [4]. The sector employs farmers and individuals working in dairy marketing and processing in the formal and informal value chains [4]. The formal value chain dominates the pasteurised milk market and has stricter compliance with food safety standards. The informal value chain transacts 86% of total milk sold, but has low food safety compliance [4]. Most studies on FSM compliance in Kenya have focused on the export and horticultural sectors due to non-tariff trade barriers associated with food safety risks [4], [21], [22]. In the dairy sector, research has mostly focused on post-farm-gate milk quality [4]. Recent studies have explored the risks of zoonoses associated with milk and milk products, such as tuberculosis and brucellosis, and foodborne pathogens such as *Salmonella* spp., *Campylobacter* spp. and Coliform bacteria (*E. coli*) at the farm level [23]. Moreover, studies have also focused on the public health impacts of poor milk quality [24], [25], antibiotic residues in milk [26], [27], [28], and aflatoxin residues in milk [29], [30]. It is therefore evident that there is a limited focus on the adoption of FSM at the farm level in smallholder dairy farms in Kenya. The main objectives of this study, therefore, were to investigate the current state of FSM adoption at the farm level and identify the drivers of this FSM adoption in the Kenyan smallholder dairy farms.

## 2 Methodology and research design

### 2.1 Study area

This study was conducted in Laikipia, Nakuru, and Nyandarua counties. These counties were selected to capture the diversity of agroecological zones and dairy systems found in Kenya, as they have a high concentration of smallholder farmers, high milk production levels, and represent the diverse dairy production systems present in Kenya [13], [31], [32].

### 2.2 Data collection

A multi-stage cross-sectional research design was employed to collect data for this study. Data on the adoption of FSM were collected using focus group discussions (FGDs) and a cross-sectional farm survey conducted sequentially. Survey respondents and focus group discussants were mutually exclusive.

### 2.3 Statement of ethics

This work had ethical approval from the International Livestock Research Institute's (ILRI) Institutional Research Ethics Committee (ILRI IREC) (REF: ILRI-IREC2017–09). IREC is accredited in Kenya by the National Commission for Science, Technology and Innovation (NACOSTI).

#### 2.3.1 Focus group discussions (FGDs)

Focus group discussions (FGDs) were used to collect qualitative data across the three counties. Smallholder farmers were recruited to participate in these discussions with the help of county extension, veterinary and livestock production officials and were purposively selected based on inclusion criteria, namely: adults (18 years and above), experience in smallholder dairy farming, and residents in the community for a minimum of 5 years.

Four FGDs were conducted per county as indicated in Table 1. Each FGD had between 6 and 9 participants. Discussions were conducted as male-only, female-only and mixed groups to avoid dominance by either gender and to allow for triangulation of male and female views. In total, 12 FGDs were conducted; 4 male-only FGDs, 4 female-only FGDs and 4 FGDs with mixed-gender participants. In total, 72 farmers, i.e. 36 males and 36 females, participated in the FGDs.

**Table 1:** Summary of focus group discussion participants.

	Laikipia	Nakuru	Nyandarua	Men	women	Total
Male FGDs	2	1	1	24	–	24
Female FGDs	1	2	1	–	24	24
Mixed FGDs	1	1	2	12	12	24
Total	4	4	4	36	36	72

FGDs were conducted using a semi-structured interview guide comprising open-ended questions. FGDs participants were asked about their knowledge and adoption of FSM, namely, related to animal health, milking hygiene, milk storage, environmental hygiene, and farming characteristics. The discussions were facilitated by a moderator, a note-taker and an interpreter who was fluent in the predominant local language in the area (Kikuyu), and the national languages, Kiswahili and English. The FGDs were held in the villages either in the morning or in the afternoon in one of the farmers' homesteads and lasted between 60 and 75 min. Clarification on emergent issues was sought from community elders, extension workers and farmer group leaders after the discussions had concluded. The FGDs were recorded using digital recorders with the prior consent of the discussants.

#### 2.3.2 Cross-sectional farm survey

A cross-sectional survey informed by the findings obtained through the FGDs and a systematic literature review was used to collect quantitative data. This survey comprised both open and closed-ended questions. Data collected related to the on-farm adoption of FSM related to animal health, milking hygiene, hygienic storage, and maintenance of hygienic premises and the surrounding environment practices [1], [6], [7], [9], [12], [13].

In this study, we used a spatial framework based on distance to markets and smallholder intensification to categorise farmers. Smallholder dairy farming systems were categorised into intensive smallholder dairy farming systems in urban locations (UL), semi-intensive smallholder dairy farming systems in mid-rural locations (MRL) and extensive smallholder dairy farming systems in extreme rural locations (ERL) [13], [31], [32], [33]. These farming locations were based on spatial proximity to a major milk market, as follows: areas within 20 km of a major town were classified as UL; those 20–45 km away as MRL; and those beyond 45 km as ERL [13].

The sample size was determined through a proportional sampling methodology for a finite population. We assumed that the proportion of participants who adopt and implement FSM is 50%. We considered a 5% precision margin and added a 15% margin to account for non-response and possible incomplete questionnaires, as has been explained by [13], [34]. This resulted in a sample size of 460 smallholder dairy farming households. To increase the external validity of the survey findings and due to the availability of resources, the researchers proportionately increased the sample size across the counties, which resulted in a final sample size of 652 smallholder dairy farming households.

In all three counties, we stratified them into UL, MRL and ERL before selecting a representative sample of smallholder dairy farmers. In each county, we interviewed 210 smallholder farmer households from the UL, MRL and ERL. Survey respondents were identified through simple random sampling. The survey was conducted with the head of each household who was male or female, aged 18 years and above; where this was not possible, we interviewed a member of the household with knowledge related to dairy production and/or the management of the farm. Approximately 79.2% (529 of the 652 participants in the survey) of the respondents were either the household head or the spouse of the household head.

Before the survey was administered, the questionnaire was pretested to test the appropriateness of questions and the duration required to answer the questions with 25 respondents with similar characteristics to farmers selected in the study sites. These farmers were excluded from the main survey. The questionnaire was amended as necessary before data collection. The survey questionnaire was administered by five trained enumerators who could speak Swahili, Kalenjin and Kikuyu and took approximately one hour to administer.

### 2.4 Data management and analysis

The recorded FGD audio data were translated and transcribed into English, and the transcripts were checked against the notes taken during the interviews to ensure consistency by a trained research assistant with a good command of English, Swahili and Kikuyu. Data were managed and coded into emergent themes, and inductive content analysis was performed with NVIVO®. The analysis involved the generation of initial codes, collation of codes into identified recurring themes and analysis using the refined themes and research objectives. Content analysis based on the research objectives led to the identification of recurring themes, similar patterns and quotations supporting findings related to the adoption of FSM.

Survey data were entered into Excel and cleaned before analysis. All statistical analyses were conducted using R version 4.2.3 statistical software (R Core Team, 2023) [35] and Stata Corp, (2022) [36]. Descriptive statistics were performed to summarise farm and farmer characteristics.

### 2.5 Empirical Frameworks

#### 2.5.1 Adoption of Food Safety Measures (FSM)

To assess the extent of the adoption status of food safety measures (FSM) in smallholder dairy farms in Kenya, we constructed a Food Safety Index (FSI) following the approach of [11]. FSM can be defined as any practice or measure implemented at the farm level to minimise food physical, chemical and microbial contamination [6], [7], [10], [11]. FSM relevant to smallholder dairy production farms in Kenya were identified through a literature review and informal discussions with subject experts within the research project. The selected FSM measures reflect established principles of Good Agricultural Practices (GAPS) [37], a customised food safety assessment applied to the Tanzania dairy sector [12] and farm-level FSM adoption studies in India and Nepal [6], [7], [10], [11]. These measures were related to the control of chemical and/or microbiological hazards and reflected farmers' implementation of animal health measures, milking hygiene and milk handling measures, milk storage hygiene and cleanliness of animal and dairy premises practices. This study considered 30 farm-level measures: 11 milking hygiene, 6 milk storage, 6 environmental hygiene and 7 animal health measures, respectively.

Data collected from smallholder dairy farmers were then used to compute weighted food safety index (FSI) ranges for each farm, as proposed by [6], [11]. The FSM adopted by dairy farmers were grouped into hygienic milking, hygienic storage, animal health, and hygienic environment/surrounding practices and assigned weights of 0.35, 0.20, 0.25, and 0.20, respectively. The FSM weights are based on their relative importance in ensuring milk safety [6], [11]. The percentage of adopted measures in each category was multiplied by the respective weight to obtain an FSI score. FSI for the “i^th^” farm was computed as explained by [6] and represented by Eq. (1):

(1) $${\mathrm{FSI}}_{i}=\sum_{j=1}^{4}\mathrm{WjPj}$$

Where: W = Weight of the j^th^ hygienic category (j takes the values of 1, 2, 3 or 4), P = proportion of practices adopted in each category adopted by farm households. The resulting FSI ranges from 0 to 100, with higher values indicating a greater farm-level adoption of FSM.

The FSI can be summarised as follows: FSI = (proportion of adopted animal health ∗ 0.25) + (proportion of adopted milking hygiene ∗ 0.35) + (proportion of adopted hygienic storage ∗ 0.20) + (proportion of adopted hygienic environment surrounding and/or practice ∗ 0.20).

For ease of interpretation and subsequent econometric analysis, the FSI scores were analysed both as a continuous and an ordered categorical outcome variable. Following previous studies [6], [11], We categorised farms into three adoption classifications based on the scores: low adopters (FSI < 30%), medium adopters (FSI 30–60%) and high adopters (FSI > 60%). These categories captured meaningful differences in levels of food safety practices and also facilitated the identification of factors associated with the adoption levels.

#### 2.5.2 Drivers of FSM adoption

The adoption of FSM is driven by different farm-level factors. To understand the relationship between the adoption levels of FSM and various farm-level drivers, we estimated both univariable and multivariable regression models. The univariable models were used to describe the crude (unadjusted) associations between the explanatory factors and FSM outcomes, while the multivariable models were used to estimate these associations while adjusting for potential confounding by farm, farmer or market level covariates.

First, we examined the relationships between farm-level characteristics and the adoption of each of the four earlier defined FSM domains using ordinary least squares (OLS) regression. For the univariable analyses, each explanatory variable was included separately into an OLS model with each FSM domain (hygienic milking, hygienic storage, animal health, and hygienic environment/surroundings) adoption percentage as an outcome variable, as specified in Eq. (2):

(2) $$Y=\mathrm{\beta o}+x^{\prime }\beta +\varepsilon$$

Where: Y is the column vector (percentage of adopted FSM), x is the vector of independent variables expected to influence the adoption of FSM, and β are parameters to be estimated. ε is the column vector of error terms, assumed to be independently and identically distributed.

We then used univariable OLS regression with the weighted FSI as the outcome variable to examine the independent drivers of overall farm-level FSM adoption, as shown in Eq. 3.

(3) $$Y=\mathrm{\beta o}+x^{\prime }\beta +\varepsilon$$

Where: Y is the column vector (farm FSI), x is the vector of independent variables expected to influence the adoption of FSM, and β are parameters to be estimated. ε is the column vector of error terms, assumed to be independently and identically distributed.

To complement the continuous FSI analysis and understand the adoption intensity, we modelled the aggregated FSI outcome ranking farmers into low, medium and high adopter categories using ordered logistic regression, as explained by [6]. The main objective of this model was to explore the factors that can be used to nudge farmers to shift to a higher FSM adoption category, as also explained by [6], [11]. As the dependent variable was categorical and ordered in nature, an ordered logit model was appropriate as specified in eq. 4 below.

(4) $${Y_{i}}^{*}=\mathrm{\beta o}+x^{\prime }\beta +\varepsilon$$

Where:

Y = 0 if Y* ≤ 0 (low adopter group),

Y = 1 if 0 < Y* ≤ μ_1_ (medium adopter group).

Y = 2 if μ_1_ < Y* ≤ μ_2_ (high adopter group).

Across both sets of regression models, multivariable analyses were conducted including the full set of explanatory variables considered theoretically relevant, including farmer demographic characteristics (e.g. age, sex, education and farmer experience), farm structural attributes like herd size and proportion of milk sold, access to resources and services, institutional factors and market participation (formal, informal or mixed value chains). To account for unobserved regional heterogeneity related to ecological conditions, infrastructure and local governance contexts, we included the county as a fixed effect in the models. Model diagnostics were performed to assess their underlying assumptions, including checking for multicollinearity, heteroscedasticity and model specifications. Robust standard errors were employed appropriately to ensure valid statistical inference.

## 3 Results

### 3.1 Farm characteristics

The characteristics of smallholder farms in the three counties are presented in Table 2. The majority of farmers were aged between 30 and 60 and practised mixed crop-livestock farming on small parcels of land. Most farmers kept Holstein-Friesian crosses; however, in dry semi-arid locations, local breeds were kept. FGDs revealed that smallholder farmers preferred Holstein-Friesian cows due to their high milk production. However, they also reported that the crossbreed required a large quantity of feed and produced milk with low density.

“*I have a crossbreed of Ayrshire and Friesian* [..] *it has good quality milk* […] *Ayrshire does have better quality milk than Friesian, but the disadvantage is the milk is not a lot like the way Friesians produce* ” FGD Mutrakwa.

**Table 2:** Farm and farmers' characteristics as a percentage (*n* = 652).

		Laikipia (32.36%)	Nakuru (33.74%)	Nyandarua (33.90%)	Total (100%)
Milker gender	Male	45.02	43.64	51.58	46.8
	Female	54.98	56.36	48.42	53.2
Education level	No formal	14.22	8.18	8.14	10.12
	Primary school	42.65	36.36	42.08	40.34
	Secondary school	32.23	43.18	38.46	48.04
	Post-secondary school	10.90	12.27	11.31	11.50
Milk marketing channels	Subsistence (only selling excess)	17.06^a^	24.09 ^c^	11.76^a^	17.64
	IVC	60.19^c^	55.45 ^c^	31.67	48.93
	FVC	20.38^a^	16.36^a^	55.66 ^b^	30.98
	IVC &FVC	2.37	4.09	0.90	2.45
Had access to animal health		98.58^a^	95.45^a^	99.55 ^b^	97.85
Had access to water		72.99^a^	89.09 ^b^	82.81 ^b^	81.75
Awareness of hygiene regulations		91.47^a^	90.91^a^	98.19 ^b^	93.56
Member of a cooperative		27.49^a^	23.64^a^	38.46 ^b^	29.91
Had attended milk quality training		26.07^a^	22.73^a^	34.84 ^b^	27.91
Farming years (Experience)	<10	13.74^a^	22.27^a^	23.08 ^b^	19.79
	10–20	28.91	28.64	33.03	30.21
	21–30	18.96	19.09	17.65	18.56
	>30	38.39 ^c^	30.00	25.79	31.29
		**X̄ (S. E)**	**X̄ (S. E)**	**X̄ (S. E)**	**Average X̄ (S.E)**
Amount of milk sold	(litres/day)	8.11 (0.67)^a^	9.56 (1.07)^a^	15.04 (2.55) ^b^	10.95 (0.97)
Milker age	(years)	47.91 (1.23) ^a^	41.76 (0.96)^b^	42.55 (0.93)^b^	44.02 (0.61)
Total number of cows		3.76 (0.22)^b^	5.53 (0.46) ^b^	4.32 (0.30)^a^	4.55 (0.20)
Number of milking cows		2.16 (0.13)^a^	2.80 (0.22) ^b^	2.30 (0.15)^a^	2.42 (0.10)
Milk price in the rainy season	(Kenya shillings/l)	30.16 (0.48)^a^	34.12 (0.47) ^b^	30.07 (0.29)^a^	31.46 (0.25)
Milk price in the dry season	(Kenya shillings/l)	36.85 (0.62)^a^	40.86 (0.62) ^b^	36.43 (0.29)^a^	38.06 (0.31)
Average market milk price	(Kenya shillings/l)	33.50 (0.48)^a^	37.49 (0.50) ^b^	33.25 (0.23)^a^	34.76 (0.25)

IVC-Informal value chains, FVC- Formal value chains. X̄ mean, S.E.- standard error of mean

Values within a row sharing the same superscript letter are not significantly different at 5% significance level. Superscripts refer to comparisons between counties only

There were differences in farms in urban and rural areas in all three counties. Farms in urban areas were characterised by intensive production, kept high-producing dairy cows, and relied more on external, purchased fodder and feed resources. Farms in the rural areas were semi-intensive and/or extensive, kept Holstein-Friesian crosses and local breeds and planted fodder.

“*I have planted fodder* [turnip, Napier grass, oats and fodder maize] *and because of some training we received from vets and extension officers, I have also added lupin*” FGD Mutrakwa.

“*All through the* dry season *I had silage, and you notice when you give silage milk is constant without going down* [no seasonal fluctuations]*. But the moment we run out of silage, and start giving cows other feeds straight from the farm, you notice a decline in milk production*”  FGD Mutrakwa.

Milk in all three counties was sold through formal and informal value chains. Almost all of the milk produced in urban and peri-urban locations entered the informal value chains. In rural areas, farmers sold to milk processors through farmer groups and cooperatives in the formal value chain, while a significant amount was also sold in the informal value chain through small-scale traders and middlemen.

Farm-gate milk prices were determined by processors in the formal value chain. Informal value chain actors paid higher farm-gate prices and sold milk at a lower retail price to consumers compared to the formal value chain. Milk prices were influenced by the prevailing environmental conditions, which influenced milk supply, and were typically higher in the dry season compared to the rainy season. Farm gate milk prices were higher in the urban areas than in rural areas.

*“Milk prices have always been low; there is no time when the prices go any higher. It goes even to as low as 24 shillings. When you consider the cost of production and the milk prices, there is not that much profit.”*  

FGD Molo

### 3.2 Adoption of FSM at the farm level

The intensity of FSM adoption at a farm level plays an important role in determining milk safety and quality. The average FSM adoption by smallholder dairy farms is presented in Table 3. The adoption of good milk handling hygiene measures at the farm level was low (Table 3). A large proportion of farmers used the same water and drying towel for all of the animals, which is a risky practice that could spread mastitis bacteria between cows. Few farmers tested for mastitis or disinfected the cow teats before and after milking. Survey and FGDs results revealed that the majority of farmers used untreated water from dams and rivers to clean milking and storage containers (e.g. milking buckets).

“*Milk and dirt do not mix* [should not mix], *you have to be clean, including during milking, by washing and drying the udder well*.” FGD Engineer.

“*That is too much work* [using different towels and water to clean udders of each cow], *it is even difficult to get firewood for warming that water* [used for cleaning]” FGD Olkalau.

**Table 3:** Summary of FSM adopted by smallholder dairy farmers in Kenya (*N* = 652) (Arranged from most adopted to least adopted FSM).

Adopted milking hygiene measures	%	Adopted milk storage measures	%	Adopted environmental hygiene measures	%	Adopted animal health measures	%
Do not use calf suckling for milking	94.5	Storage Containers cleaned properly with water, soap/disinfectant well	95.6	Cattle housed (not extensive outdoors)	50.2	Cattle regularly dewormed	98.9
Wash your hands before milking	93.4	Use recommended containers for storage, i.e. Mazzican or aluminium containers	44.3	Clean sleeping/resting area	38.3	Controls ectoparasites, e.g., ticks	91.2
Wash udder and teats before milking	93.4	Controls flies in the milk storage area	28.4	Clean the cow shed daily	33.7	Vaccinates cows	83.1
Dry the udder and teats after washing	89.7	Test milk for quality before storage	26.5	Regular cleaning of the urine trench/ trough	23.1	Regular cleaning of the water trough	68.2
Dry your hands after washing them	78.7	Use treated water to clean Water	19.1	Regular cleaning/emptying of the manure/urine pit	22.5	Regular cleaning of the feed trough	50.5
Tying the cow's legs and tail (restricting movement) during milking	61.2	Discard poor quality	6.7	Cleaning cow dung if cows defecate during the day to keep the shed clean	0.6	Tests cows for mastitis	
Observe the withdrawal period for treated animals	55.8					Cattle provided with clean drinking water	22.1
Clean the milking area before milking cows	52.6						
Use separate udder towels for each cow, cleaning and drying	30.5						
Disinfect teats before and after milking	25.6						
Use treated water for cleaning milking containers and equipment	22.1						

The majority of farmers used non-food-grade plastic containers for milking and milk storage, which was contrary to the food standards recommendations.

“*I use plastic milking containers; they are hung outside the house* [..] *I wouldn't like to lie that I use metal one*s” FGD Mutarakwa.

“*I milk with an aluminium bucket and then pour the milk into a plastic container*” FGD Molo.

“*The cost of the recommended containers* [stainless steel and aluminium] *is too high. If you tell me to go buy a container for five thousand and the same in plastic is one hundred, I will opt for the cheaper one due to the economic issues*” FGD Olkalau.

The adoption of good milk storage practices at the farm level was low (Table 3). There was low testing of milk for quality before storage; this mainly occurred in big farms. The low percentage of farmers who discarded milk from treated animals reflected low knowledge of milk quality standards and regulations, disregard for standards, and greed (unwilling to forego lost income). Some farmers expressed misconceptions and assumed that mixing milk during the bulking process eliminated the risks posed by antibiotics. Farmers in all three counties reported that animal health practitioners did not emphasise the need for observing the withdrawal periods; and that unscrupulous para-vets gave incorrect advice to farmers, for example, they advised farmers to treat several animals at a time and mix the milk of treated and untreated cows to avoid detection, instead of completely discarding the milk. FGD results showed that the majority of farmers strived to pass the minimum tests done by buyers, namely, density and alcohol tests, which do not detect antibiotics and aflatoxin contamination in milk.

“[The only test during milk bulking] *It's only density and if the milk has any smell. They use an alcohol gun”* FGD Olkalau.

FGD discussions revealed that the adoption of good environmental measures was not adhered to by the majority of farmers who practised zero-grazing. Cows were kept in housing that had an earthen floor, which became muddy in the rainy season and was also difficult to clean. In extensive farms, where cows were grazed and milked in open fields, it was difficult to control contamination due to exposure to vectors such as flies and environmental contaminants. FGDs revealed that, in urban areas, disposal of manure was a problem as the farm sizes were small, which led farmers to release slurry into canals or pile it until there was a quantity large enough to move to another farm or be sold. The lack of regular cleaning of cowsheds could reflect competition for labour between crop farming activities, dairy husbandry activities, and household chores. Most farmers could only clean their cowsheds once every few days.

The adoption of animal health practices in smallholder farms was high. Although farmers said they checked for mastitis, FGD discussion revealed that the majority of farmers did not conduct milk stripping tests to check for mastitis, while only a small percentage of farmers performed teat dipping. There was good control of ectoparasites on farms through regular dipping or injection with broad-spectrum antihelminth drugs such as ivermectin. FGDs revealed, however, that farmers rarely observed a withdrawal period after treating their animals and only a few farmers discarded milk from treated sick cows and treated cows or fed it to calves in some cases, particularly where the smell of drugs could be detected by organoleptic measures.

*“That is where we have a challenge because, as I told you I'm milking 10kgs and if I give it antibiotics, the vet says I should not consume it, nothing about not selling it if I have ever been told to pour it or give dogs, then that would be another case”* FGD Molo.

*“Sure, you're told not to drink it for 72 hours because of the antibiotics, and even we humans, after being treated, feel the urine smelling of drugs. The issue is that the poverty level is making farmers disregard some of these things. It presses us to do things you know you shouldn't”* FGD Molo.

*“I think there is no harm, the milk will be mixed with other milk, and it will neutralise the antibiotics, and also the processing will take care of it with boiling, pasteurisation and whatever else they do in the processing plants, and I'm the only person who knows I had given the antibiotics.”* FGD Molo.

Table 4 presents FSI, which describes the intensity of FSM adoption. There were significant differences in environmental hygiene measures between the counties, with Nakuru County having the lowest adoption rates. There were also significant differences in animal health-related FSM adoption between the counties, with Nyandarua having the lowest rate of adoption. The FSI of each farm was used to categorise smallholder dairy farmers as follows: 8.9%, 57.1% and 33.89% as low, medium and high adopters of FSM, respectively.

**Table 4:** weighted FSM in smallholder dairy farmers (*N* = 652).

	Laikipia		Nakuru		Nyandarua		Average	
FSM	X̄	S. E	X̄	S. E	X̄	S. E	X̄	S. E
Milk handling hygiene	16.77	0.35	16.85	0.30	16.77	0.31	16.80	0.18
Milk storage measures	7.16	0.23	7.33	0.24	7.68	0.20	7.39	0.13
Environmental hygiene measures	23.21^b^	0.39	20.07	0.44	23.34^b^	0.32	22.19	0.23
Animal health measures	6.48^c^	0.38	6.35^c^	0.44	3.92	0.37	5.57	0.24
Food safety index (FSI) %	53.35	1.00	50.22	1.09	51.47	0.83	51.65	0.57
FSI Range	13.66–90.15		13.66–80.45		20.32–83.25		13.66–90.15	

Note: Values not sharing the same superscript are significantly different at *p* < 0.05

FGDs revealed that there were differences in access to inputs, capital and milk quality technology. Farmers in rural areas were at a disadvantage compared to their peers in urban and peri-urban areas in their access to technology. Input suppliers, extension providers and financial institutions were located in urban areas, which reduced transaction costs in urban areas relative to rural areas. The quality of road infrastructure in rural areas constrained the timely delivery of milk from farms to milk cooling plants and markets, especially in the rainy season.

FGDs revealed that the current payment system prevailing in the Kenyan dairy sector does not incentivise farmers to invest time and resources in realising milk quality improvements. The current payment system rewards farmers based on volume, rather than the quality of milk. The sanctioning of farmers for poor milk quality is ineffective, as milk rejected in the formal value chain is often sold by unscrupulous traders in the informal value chain. Lack of investment and ineffective sanctioning were reported by farmers as barriers to investing in milk quality improvement technologies and measures, such as improved milking equipment and containers, as the incurred costs would not be compensated by milk prices. Farmers believed that effective interventions beyond the farm level were needed, as contamination and adulteration also occurred in the value chain.

*“We sell it* [rejected milk] *there is no need to lie to you”* FGD Molo.

Table 5 presents the results of the univariable OLS regression conducted to explore the drivers underpinning the adoption of the four categories of FSM adoption. The data were tested for reliability before regression models were performed (see appendix). Both models suggest that farmers in Nakuru and Nyandarua were less likely to adopt milk hygiene and environmental and animal health FSM than in Laikipia. Participation in the formal value chains was associated with high adoption of FSM. When the milker was on a farm, it was associated with more adoption of FSM. Farmers with post-high school education had higher adoption rates of milking hygiene and animal health FSM. Highly experienced farmers were less likely to adopt milking hygiene FSM. Having access to clean and treated water was associated with increased adoption of all FSM. Knowledge of milk quality standards and hygiene regulations was associated with high adoption of all FSM, except for environmental FSM. Cooperative and farmer group membership, which is a proxy for access to information and financial resources, was associated with low adoption of animal health and milk storage FSM. Access to animal health was associated with low adoption of milking and environmental hygiene FSM.

**Table 5:** Univariable OLS regression models of farm-level factors associated with adoption of FSM domains (*n* = 652).

	Milk hygiene	Environmental hygiene	Animal health	Milk storage
(Intercept)	31.89 *** (5.54)	8.97 (12.46)	34.07 *** (7.56)	19.92 *** (6.49)
County Nakuru	−9.03 *** (1.33)	−4.08 (2.98)	−1.51 (1.81)	−0.54 (1.55)
County Nyandarua	−3.24 ** (1.35)	−16.34 *** (3.03)	−3.62 ** (1.84)	−1.38 (1.58)
Marketing channel- informal value chain	5.37 *** (1.69)	7.41 * (3.79)	3.06 (2.30)	0.54 (1.98)
Marketing channel- formal value chain	7.80 *** (1.98)	10.34 ** (4.46)	6.80 ** (2.70)	5.85 ** (2.32)
Marketing channel- both informal & formal value chain	−2.04 (3.71)	−4.67 (8.36)	0.44 (5.07)	6.24 (4.36)
Proportion of milk sold	−4.01 (3.33)	5.39 (7.50)	1.54 (4.55)	8.04 ** (3.91)
Milker age	0.03 (0.04)	−0.04 (0.08)	0.00 (0.05)	−0.05 (0.04)
Milker gender	3.04 *** (1.05)	6.35 *** (2.37)	2.12 (1.44)	0.32 (1.23)
Education level- primary school	0.39 (1.84)	−5.64 (4.15)	2.99 (2.52)	−0.42 (2.16)
Education level- secondary school	−0.10 (1.92)	−2.74 (4.33)	3.55 (2.63)	0.37 (2.26)
Education level- post-secondary school	4.37 * (2.31)	4.44 (5.19)	6.81 ** (3.15)	2.64 (2.71)
Experience 10–20 years	1.17 (1.48)	0.49 (3.33)	1.29 (2.02)	−5.48 *** (1.74)
Experience 21–30 years	1.69 (1.68)	0.21 (3.78)	−1.30 (2.29)	−5.25 *** (1.97)
Experience >30 years	−2.84 * (1.62)	−2.09 (3.66)	−1.40 (2.22)	−1.20 (1.91)
Total number of cattle	0.01 (0.11)	−0.04 (0.24)	0.24 (0.15)	0.18 (0.13)
Having access to treated piped water	2.95 ** (1.34)	10.74 *** (3.02)	8.09 *** (1.83)	4.38 *** (1.58)
Knowledge of hygiene regulations	36.83 *** (2.20)	9.48 * (4.94)	18.26 *** (3.00)	15.40 *** (2.58)
Cooperative membership	−1.35 (1.31)	0.94 (2.95)	−4.26 ** (1.79)	−3.53 ** (1.54)
Has had training in milk quality	−0.19 (1.21)	1.96 (2.72)	−0.04 (1.65)	3.86 *** (1.42)
Average milk price	0.02 (0.08)	0.39 ** (0.18)	−0.06 (0.11)	−0.08 (0.10)
Access to animal health	−6.99 ** (3.51)	−15.18 * (7.89)	3.55 (4.79)	−1.40 (4.11)

Education base- no formal education, milk marketing channels- subsistence.

*** *p* < 0.01; ** p < 0.05; * *p* < 0.1.

### 3.3 Empirical results of models relating to factors associated with FSM adoption

The results of the univariable OLS and ordered logistic regression estimated coefficients and standard errors are presented in Table 6. The results of the empirical analysis provide information about the relationship between farm-level adoption of FSM and the characteristics of smallholder dairy farmers and are not adjusted for potential confounding by other factors. The dependent variable for the OLS regression is the continuous FSI developed for the weighted FSM, while the ordered logistic coefficients represent the changes in log-odds of being in a higher FSM adoption category.

**Table 6:** Univariable regression models of Farm-level factors associated with milk FSM adoption.

	OLS (FSI) model	Ordered logistic model
(Intercept)	25.46 *** (5.34)	
County Nakuru	−4.46 *** (1.28)	−0.42 (0.28)
County Nyandarua	−5.58 *** (1.30)	−0.69 ** (0.29)
Marketing channel- informal value chain	4.23 *** (1.63)	0.84 ** (0.39)
Marketing channel- formal value chain	7.67 *** (1.91)	1.59 *** (0.45)
Marketing channel- both informal & formal value chain	−0.29 (3.58)	−1.11 (0.90)
Proportion of milk sold	1.67 (3.21)	0.74 (0.73)
Milker age	−0.01 (0.03)	−0.00 (0.01)
Milker gender	2.93 *** (1.02)	0.51 ** (0.23)
Education level- primary school	−0.33 (1.78)	−0.42 (0.40)
Education level- secondary school	0.38 (1.86)	−0.19 (0.41)
Education level- post-secondary school	4.65 ** (2.23)	0.51 (0.46)
Experience 10–20 years	−0.27 (1.43)	−0.33 (0.30)
Experience 21–30 years	−0.74 (1.62)	−0.42 (0.35)
Experience >30 years	−2.00 (1.57)	−0.73 ** (0.34)
Total number of cattle	0.09 (0.10)	0.02 (0.02)
Having access to treated piped water	6.08 *** (1.30)	0.98 *** (0.31)
Knowledge of hygiene regulations	22.43 *** (2.12)	4.21 *** (0.48)
Cooperative membership	−2.06 (1.26)	−0.41 (0.28)
Has had training in milk quality	1.09 (1.17)	0.16 (0.25)
Average milk price	0.05 (0.08)	0.00 (0.02)
Access to animal health	−4.88 (3.38)	−0.66 (0.70)

Education base- no formal education, milk marketing channels- subsistence

*** p < 0.01; ** p < 0.05; * p < 0.1.

The results of the OLS model reveal that farmers in Nakuru and Nyandarua were less likely to adopt FSM compared to farmers in Laikipia, as there were no perceived incentives at the county level to encourage FSM adoption by smallholder farmers. Participation in formal and/or informal marketing channels was associated with increased uptake of FSM. The gender of the milker was associated with the uptake of FSM, with female workers likely to adopt more FSM. There was a higher uptake of FSM at the farm level among those who had post-high school education. Finally, farmers' knowledge of milk quality regulations and access to treated piped water were positively associated with the increased adoption of FSM.

The dependent variable for the ordered logistic regression was a discrete ranked outcome of the intensity of adoption that ranked farmers as low, medium and high adopters. The results of the ordered logistic regression also reveal that farmers in Nakuru or Nyandarua were less likely to adopt FSM compared to farmers in Laikipia. Participation in formal or informal marketing channels was associated with increased uptake of FSM. The gender of the milker positively influenced the adoption of FSM; for example, female milkers had a higher adoption of FSM than men. Experienced farmers were likely to have lower adoption of FSM. Post-high school education level was associated with higher uptake of FSM at the farm level. The magnitude of adoption was smaller according to the ordered logistic model compared to the OLS model. All the significant predictor variables were similar except for farming experience, which was a significant variable in the ordered logistic model but not in the OLS model. Further model diagnostics and refitting showed that the multivariable OLS specification for the continuous FSI scores had a better fit and provided a more straightforward interpretation of the adjusted mean differences than the ordered logistic model.

#### 3.3.1 Adjusted association between milk marketing channel and FMS adoption

In the final adjusted OLS model (Table 7), the association between marketing channel and FSI scores was not constant across farms; rather, it varied jointly by county and household milk consumption, indicating a statistically significant effect modification. A block test for the three-way interaction between marketing channel, amount of milk consumed in the household and county was highly significant (*p* < 0.001), demonstrating that the association between marketing channel and FSI scores changes with household milk consumption and is specific by county. As such, the interaction coefficients are interpreted as conditional associations. However, there was no statistically significant evidence that household head education level or sex modified this association.

**Table 7:** Multivariable (Adjusted) Model of Factors Associated with FSM Adoption.

Predictor	Coefficient	Robust SE	t	*P*-value	95% CI	
Main effects					Lower	Upper
Marketing channel: Formal market	3.648	3.122	1.17	0.243	−2.483	9.779
County: Nakuru	−5.489	2.370	−2.32	0.021	−10.143	−0.836
Nyandarua	−0.804	2.256	−0.36	0.722	−5.235	3.627
Milk consumption (per 1-unit increase)	0.992	0.360	2.75	0.006	0.284	1.700
Education: Secondary	1.996	2.171	0.92	0.358	−2.266	6.258
Tertiary	3.993	2.149	1.86	0.064	−0.227	8.212
Household head sex: Male	4.628	1.108	4.18	0.000	2.453	6.803
Access to water: =1	5.637	1.317	4.28	0.000	3.050	8.224
Two-way interaction terms						
Formal market × Nakuru	10.945	4.968	2.2	0.028	1.190	20.701
Formal market × Nyandarua	−0.753	3.936	−0.19	0.848	−8.482	6.976
Formal market × Milk consumption	0.705	0.738	0.96	0.340	−0.745	2.155
Nakuru × Milk consumption	0.803	0.688	1.17	0.244	−0.549	2.154
Nyandarua × Milk consumption	−1.142	0.513	−2.23	0.026	−2.148	−0.135
Three-way interaction						
Formal market × Nakuru × Milk consumption	−4.286	1.115	−3.84	0.000	−6.476	−2.097
Formal market × Nyandarua × Milk consumption	−0.199	0.905	−0.22	0.826	−1.975	1.578
Constant						
Intercept	40.526	2.807	14.44	0.000	35.013	46.038

**Reference categories:** Informal market (channel); Laikipia (county); Primary (education); hh_sex = female; access_water = 0.

Accordingly, the final model adjusted for county, household milk consumption, sex of household head and access to water, while retaining the hierarchical set of lower-order terms used in the significant three-way (joint) interactions. This interaction indicates that the association between amount of milk consumed in households and FSI scores differed across counties, and that this county-specific slope further differed depending on whether farmers market their milk through formal versus informal markets. In this model, after adjusting for this effect modification, sex and access to water remained strong independent predictors of FSI: male-headed households (β = 4.628, *p* < 0.001) and households reporting access to water (β = 5.637, p < 0.001) had higher FSI scores. Household milk consumption also showed a positive association with FSI scores in the reference county (Laikipia) and marketing (informal) channel, but this association differed by county and marketing channel, as demonstrated in the visual figures below. Although the education level of the household head did not meet the conventional statistical significance threshold in the final model, earlier main effect fitting and tertiary education level in the final model showed a borderline positive association relative to primary education, hence retained in the final model.

**Fig. 1:**
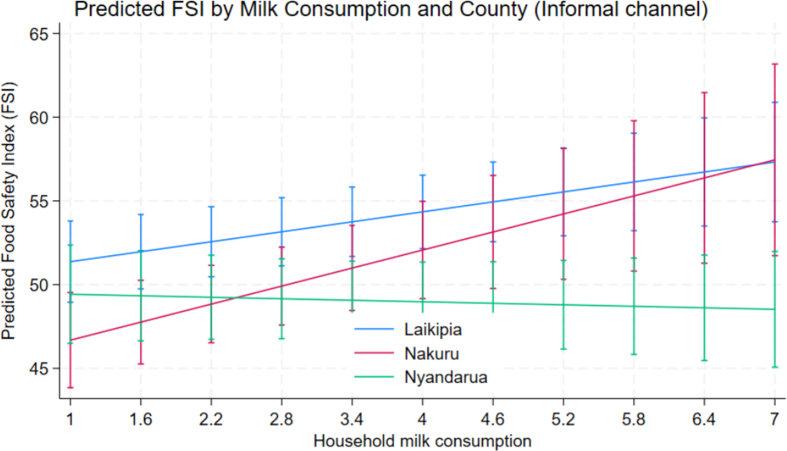
Predicted FSI by milk consumption and county (Informal marketing channel).

**Fig. 2:**
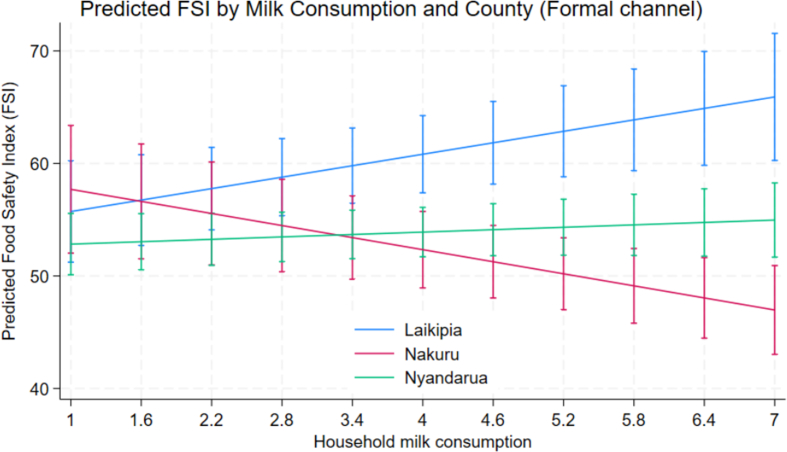
Predicted FSI by milk consumption and county (Formal marketing channel).

We established a key substantive finding regarding heterogeneity in the marketing channel association by level of household milk consumption and the farmer's county. Specifically, the joint term for Nakuru county was negative and statistically significant (β = −4.286, *p* < 0.001), suggesting that, in Nakuru, an increase in household milk consumption was associated with a reduction of FSI scores between those marketing formally versus informal marketing channels, relative to the reference county (Laikipia). This pattern is visually evident in the milk marketing channel-specific interactions plots^1^ Fig. 1, Fig. 2, where the slope for Nakuru county increases with milk consumption under the informal markets but reverses and declines under formal milk markets. The corresponding term for Nyandarua was small and not statistically different from Laikipia county's, reinforcing that the milk-consumption slope in FSI scores differs by county even before adjusting for marketing channel-specific modification. In both figures (below), the Nyandarua county trajectories remain relatively flat across milk consumption levels, indicating limited sensitivity of FSI scores to milk consumption regardless of the milk market.

We observe that formal market structures impose stricter quality standards and provide better access to training and resources. There is geographical heterogeneity, which significantly modifies several relationships that could potentially indicate regional variations in milk quality training program effectiveness, likely due to differential extension service quality. The visual plots (Fig. 1, Fig. 2) show that Laikipia county consistently maintains higher predicted FSI scores across household milk consumption levels – especially those marketing under formal markets – demonstrating that formal market gains in FSI are not homogenous across counties.

Large-scale producers in Laikipia exhibited higher FSI scores given the negative coefficients for Nakuru and Nyandarua, indicating that production scale advantages are geographically mediated, likely due to better access to technology and market infrastructure in certain regions. Also, the divergence between Laikipia and Nakuru widens at higher levels of milk consumption for those who market in formal channels, suggesting a strong geographic effect modification of the formal-channel association across household milk consumption intensity.

#### 3.3.2 Final OLS model specification

The statistical integrity and robustness of the final multivariable OLS model were assessed through a series of diagnostic tests. Multicollinearity was evaluated using Variance Inflation Factors (VIFs), with a mean VIF of 6.37, with the largest values concentrated among three-way interaction terms. This is expected in models that include higher-order interaction structures, because the product terms are basically correlated with their constituent variables. Importantly, the covariates included for adjustment demonstrated minimal collinearity (sex VIF = 1.06); access to water VIF = 1.05), suggesting that the interaction multicollinearity was not driven by main effect predictors.

The Breusch-Pagan test confirmed the presence of heteroscedasticity (χ^2^(1) = 9.34, *p* = 0.0022), justifying the use of Huber–White robust standard errors to ensure accurate estimation. Furthermore, a linktest was conducted to assess model specification, which indicated no significant omitted non-linear relationships or systematic patterns in the errors (_hatsq: *p* = 0.600), supporting the chosen linear functional form. Analysis of leverage and studentised residuals confirmed that no outliers exerted undue influence on the model results. Collectively, these diagnostics confirmed that the model's assumptions were reasonably met and that the presented results are stable and reliable.

## 4 Discussions

This study investigated the extent of FSM adoption in Kenyan smallholder dairy farms and the factors driving it. Findings indicate low adoption levels, which increases the risk of milk contamination across the three counties. Consumers in Kenya are increasingly demanding and willing to pay for safer food [23]. Moreover, improved food safety is becoming a precondition for smallholder access to export and high-value domestic markets [38]. Both the model results and FGD discussions indicate that farmers who are more educated, have larger herds, and have access to capital are more likely to invest in dairy equipment and practices that improve their FSI scores. Knowledge of milk hygiene regulations also increases FSM adoption. Low adoption, however, poses significant risks to food safety and livelihoods, including an increased likelihood of milk contamination and rejection. There is therefore an imperative for value chain actors and policymakers to ensure the production of safe, high-quality milk and dairy products [4].

Our findings are consistent with previous studies that report low FSM adoption among smallholder farmers [4], [21], [22]. Herd and farm sizes were similar to those reported in previous studies in Kenya [9], [27], [28], [29]. It is worth acknowledging that the widespread practice of boiling milk before consumption in Kenya mitigates some of the public health risks at the household level, particularly microbial risks. However, it does not negate the need for improved milk quality in dairy value chains [4], [8], [39]. Beyond boiling, there is a risk of milk recontamination due to poor hygienic handling and storage practices at the household level. Moreover, some communities, although a minority, still consume raw milk and dairy products, which is a public health risk [4], [39], [40]. Additionally, contaminants such as aflatoxins, heat-stable toxins such as the enterotoxins produced by *Staphylococcus aureus*, heat-resistant spores produced by *Clostridium perfringens* and *Bacillus* spp. and antibiotic residues can persist after boiling or pasteurisation [4], [8].

### 4.1 Status of FSM adoption in smallholder farms

Adoption of FSM on Kenyan smallholder dairy farms (51.67 FSI score) was lower compared to smallholder farms in Nepal (64%) [6], [7] and Ethiopia (59.67%) [14], but higher than the reported adoption for India (48%) [11]. Low adoption of FSM could compromise food safety and expose consumers of milk and dairy products to foodborne diseases risks [41]. Currently, the low adoption of FSM in smallholder dairy farms in Kenya is not in line with established norms of good agricultural practice (GAPs) and the Kenya Bureau of Standards (KEBS) regulations and has been cited as the main cause of poor milk quality in dairy value chains [8], [40]. There is therefore an imperative for supporting smallholder farmers to widely adopt FSM, as compliance at the farm level is important for guaranteeing food safety and milk quality [6], [7].

Findings of this study reveal that the majority of farmers used untreated water to clean their utensils and wash their hands, which could lead to milk contamination [40]. The extensive use of non-food-grade plastic containers by the majority of farmers for milking and storage leads to microbial contamination [13], [23], [40]. Plastic containers are difficult to clean and retain a high microbial load compared to aluminium ones, even after cleaning with disinfectants [42], [43]. There is an imperative for the wider dairy sector to create a conducive environment and assist dairy farmers in accessing recommended milking equipment and storage containers, which are currently inaccessible due to their higher costs [44].

Findings of this study reveal low adoption of environmental-related FSM, which poses a risk for milk quality and animal welfare. The adoption of cattle-shed hygiene, for example, can reduce disease incidents such as mastitis and also improve animal welfare by rendering cows less susceptible to diseases [45]. Improved housing with easy-to-clean concrete floors and regular cleaning can significantly reduce mastitis incidence and enhance the quality of milk [45], [46]. The implementation of recommended biosecurity measures and improved housing can protect cows from weather elements (e.g strong sun and rain) and also reduce the prevalence of vectors such as flies that can transmit livestock diseases [6], [7], [45], [46]. There is therefore a need for stakeholders and policymakers in the dairy sector to educate, train and facilitate farmers to access financial resources that can enable them to adopt improved housing, biosecurity measures and improved animal welfare practices.

The results of this study suggest there is a limited testing rate of milk for quality before storage, and the use of non-food-grade containers for milk storage further hampers milk quality control at the farm level [39], [40]. Unhygienic milk handling has been shown to increase microbial contamination [23], [40]. Low adoption of hygienic milking and storage measures and lack of temperature control during storage, bulking and marketing lead to poor milk quality [42]. Hygienic milking, handling and storage measures are crucial for ensuring that milk and dairy products are safe for human consumption [8], [9]. There is therefore a need to improve farmers' knowledge on hygienic milking, handling and storage measures to ensure milk quality is maintained and food safety standards are complied with.

Findings of this study reveal there was a high rate of adoption of good animal health practices, including vaccination, deworming and ectoparasite control, which shows that farmers are actively managing animal health and controlling disease risks. Disease prevention and control play an important role in ensuring food safety, as sick animals can shed infectious pathogens through their milk, which can pose human health risks, especially if the pathogens are zoonotic [46], [47], [48]. Previous studies have reported that the adoption of internal and external biosecurity measures reduces disease risks at the farm level and can contribute to improving animal health and consequently food safety [46]. There is a need, therefore, to educate farmers on biosecurity measures and the importance of FSM adoption as a way of improving the food safety of animal-source foods, particularly milk and dairy products [45], [48], [49].

### 4.2 Drivers of FSM adoption

The results of this study show that the adoption of FSM at the farm level is influenced by several factors, including socio-economic and cultural factors [9]. Socio-economic factors such as age, education, gender, access to credit services, information, labour and technology have been reported to influence the adoption of FSM [4]. Previous research has highlighted that there is a correlation between poor farmers' understanding of milk contamination pathways and the presence of risky food safety practices at the farm level [17]. Farmers' knowledge, attitudes and milk-handling practices play an important role in determining milk quality [13], [17], [50]. Ledo et al. (2021) showed that training related to FSM provided to milk handlers in the Tanzania dairy sector directly was associated with improvement of their knowledge, adoption of FSM practices and willingness to adopt FSM [47]. The FSM adopted at the farm level tends to be those practices that are socially and culturally acceptable, and perceived as economically viable by farmers [51]. Improving farmers' knowledge, attitudes and practices through training can therefore support the adoption of FSM aimed at improving milk quality [13], [17], [50].

Findings of this study reveal that farm level FSM adoption is associated with biophysical, technological, institutional and political factors [8], [9]. Factors such as the quality of road infrastructure, distance to milk cooling plants and the prevailing ecological conditions influence milk quality [21]. For example, farmers in rural areas have limited access to good road infrastructure, milk cooling and farm inputs compared to those in urban and peri-urban areas [21]. There is therefore an imperative for policymakers to invest in infrastructure such as road networks, milk cooling plants and increase access to dairy technology such as aluminium milking and storage containers, and improve access to animal health services [4], [21], [22].

The results of this study show that participation in the formal value chain can lead to a higher FSM adoption. Research has shown that formal market structures impose stricter quality standards and provide farmers with better access to training and financial resources [21], [22]. Previous research has reported that empowering value chain actors, particularly cooperatives and processors, to work with farmers can lead to improved milk quality across the value chain [22]. However, these formal structures, including contractual relationships, are not well established between dairy sector stakeholders, which constrains the improvement of milk quality and safety in Kenya [4], [8], [22]. There is a need for better value chain integration and coordination, and a transparent governance structure to ensure dairy sector-wide milk quality and safety improvement [8], [21], [22]. Another alternative approach would be to increase milk quality demands in the dominant informal value chain to ensure farm and value chain compliance with milk quality standards [4], [22]. There is an imperative for processors to provide economic incentives, such as price premiums for good quality milk or penalties for poor milk quality, and other non-economic incentives, such as the provision of food safety training and information to ensure improved farm-level milk safety and quality [8]. Previous research has reported that the economic contribution of the dairy sector to the Kenyan economy can be increased through a constructive, incentive-based approach that increases farmers' willingness to comply with food safety standards [21], [22].

### 4.3 Implications of this study

Food safety from farm to table starts at the individual farm level and is governed by private and public standards [1], [40]. The quality of manufactured dairy products also depends on the quality of raw milk and can affect the taste, shelf life and processing characteristics [40], [42]. Given that milk quality determines the quality and safety of processed dairy products, this study suggests that farmers should be encouraged to adopt FSM at the farm level to ensure that only quality milk and dairy products reach the dairy value chains and consumers [8], [40]. There is a need to ensure that the market rewards dairy producers who adopt high levels of FSM that lead to safer milk [6], [7]. Ensuring that farmers are facilitated to improve milking and handling hygiene practices and that infrastructure challenges are addressed, i.e. poor quality of road networks, lack of milk collection and storage infrastructure and prevalence of endemic zoonoses, can ensure that public health risks for consumers are reduced [3], [8], [13]. Moreover, there is a need to ensure that counties (devolved local governments) in Kenya invest in farmer training programs, provide quality animal health and extension services to increase farmers' knowledge, awareness and increase on-farm FSM adoption [8], [9], [26], [27], [40]. Moreover, supporting dairy production intensification through increased access to capital resources, market integration and coordination may support on-farm FSM adoption. The national and county governments can support processors who incentivise farmers' adoption of FSM with tax breaks and rebates for their investment in the dairy sector, including training farmers and providing milk cooling plants close to farms to maintain milk quality [8], [9], [40], [42], [43].

This paper advocates for the recognition and promotion of the One Health approach as a means of addressing food safety risks in dairy value chains. One Health is a holistic, multi-sectoral and collaborative approach that takes into consideration the complexity of the multiple food safety risks and bridges human, environment and animal health [17], [19], [50], [51]. The successful implementation of One Health will, however, depend on the strength of collaboration among researchers, industry, stakeholders, national agencies, and political officeholders and adequate funding of health activities [17], [18], [19]. There is a need for investment in adaptive, holistic, and progressive approaches to detect, prevent, monitor and control food safety risk at farm and value chain levels [19]. Moreover, there is a need for policymakers to recognise and appreciate the interconnections that exist among species, ecosystems and human society [17], [19]. Finally, there is a need for the development and deployment of good agricultural practices tailored to dairy farmers' specific needs to ensure safe food for the consumers, especially in low-income countries where regulation enforcement may be low and/or lacking [17], [50], [51].

There are opportunities for further research, especially the need to correlate FSM weights with real farm-level milk testing data to ensure that FSM interventions reflect the contexts that farmers face every day, depending on their farm size, climatic, biophysical, social and economic, market and institutional factors. This research can be particularly helpful for ensuring tailored context-specific interventions while at the same time contributing to addressing the paucity of milk quality research in the wider East Africa region.

Theoretically, this paper contributes to the current literature on on-farm FSM adoption as a sustainable approach to food safety and quality challenges in Kenya and similar countries in the wider East Africa. The qualitative approach to investigating FSM adoption complements quantitative farm-level food safety testing, which can be expensive and requires well-established laboratories and testing infrastructure, which are currently absent in emerging economies. Empirically, we show that farming typologies can be a good approach to cluster and understand farmers' behaviour, which can inform agricultural policies. However, there is a need for contextual understanding of farmers' individual needs, which may differ significantly from those of the wider farming community. The modelling approach selected for analyses should therefore consider these individual and farm-specific nuances.

## 5 Conclusion

In Kenya and the wider East African region, there have been significant interventions in the dairy chain related to improving farm production and milk quality through innovation focused on post-farm gate interventions, mainly within the value chain [1], [40]. There have been significant efforts to improve milk quality, but those have so far focused on the formalisation of the milk value chains [22]. There is therefore a need to ensure that an increase in milk production is accompanied by the adoption and implementation of FSM at the farm level to ensure improved food safety and livelihood security.

This study contributes to the literature on drivers of FSM adoption at the farm level in low-income countries by providing a case study of smallholder dairy farm systems in Kenya. The findings reveal low adoption of FSM measures that could compromise food safety and expose consumers to public health risks. There is an imperative to train farmers to improve their knowledge of FSM and food safety risks. Equally, national and county governments should enact policies that enable farmers to access capital to invest in innovations and technologies that improve food safety. Improving food safety will enable farmers to access lucrative markets that demand high compliance with food safety standards and regulations.

Overall, this study supports the conclusion that the choice of marketing milk through formal channels is associated with increased FSM practices in a manner that is context-dependent: the association between marketing channel and FSI scores varies systematically across counties and along the magnitude of household milk consumption. This geographic and behavioural heterogeneity is consistent with the possibility that market governance, local enforcement, and household-level incentives to ensure milk safety differ by setting. However, the statistical interpretation remains associational: the fitted coefficients quantify adjusted mean differences in FSI scores and their modification by observed covariates, rather than establishing causal effects; and hence, we recommend further research.

## CRediT authorship contribution statement

**Ndungu Nyokabi:** Writing – review & editing, Writing – original draft, Validation, Methodology, Investigation, Formal analysis, Data curation, Conceptualization. **Emmanuel Muunda:** Writing – review & editing, Writing – original draft, Validation, Methodology, Formal analysis, Data curation, Conceptualization. **Henrietta Moore:** Writing – review & editing, Supervision, Resources, Funding acquisition, Conceptualization. **Luke Korir:** Writing – review & editing, Writing – original draft, Validation, Methodology, Formal analysis, Data curation, Conceptualization. **Asaah Ndambi:** Writing – review & editing, Writing – original draft, Supervision, Methodology, Investigation, Formal analysis, Data curation, Conceptualization. **Charles Omanga:** Writing – review & editing, Writing – original draft, Formal analysis, Conceptualization. **Lilian Korir:** Writing – review & editing, Writing – original draft, Visualization, Validation, Methodology, Formal analysis, Conceptualization. **Lisette Phelan:** Writing – review & editing, Writing – original draft, Validation, Supervision, Methodology, Formal analysis, Data curation, Conceptualization.

## Ethics approval

This work had ethical approval from the International Livestock Research Institute's (ILRI) Institutional Research Ethics Committee (ILRI IREC) (REF: ILRI-IREC2017–09). IREC is accredited in Kenya by the National Commission for Science, Technology and Innovation (NACOSTI).

## Declaration of generative AI and AI-assisted technologies in the writing process

During the preparation of this work, the author(s) did not use any AI and AI-assisted technologies.

## Financial support statement

Data used in this study was generated under a PhD scholarship as part of the project “Local and International business collaboration for productivity and QUality Improvement in Dairy chains in SE Asia and East Africa (LIQUID)”. This project is supported by the 10.13039/501100003246Netherlands Organisation for Scientific Research‘s (NWO) Science for Global Development department (WOTRO) through the Food and Business Global Challenges Programme (GCP) (NOW-WOTRO project number W.08.250.204).

Funding and support for writing this paper were provided by the Institute for Global Prosperity, 10.13039/501100000765University College London.

## Declaration of competing interest

The authors would like to state that there was no conflict of interest resulting from funding or otherwise.

## Figures and Tables

**Figure:**
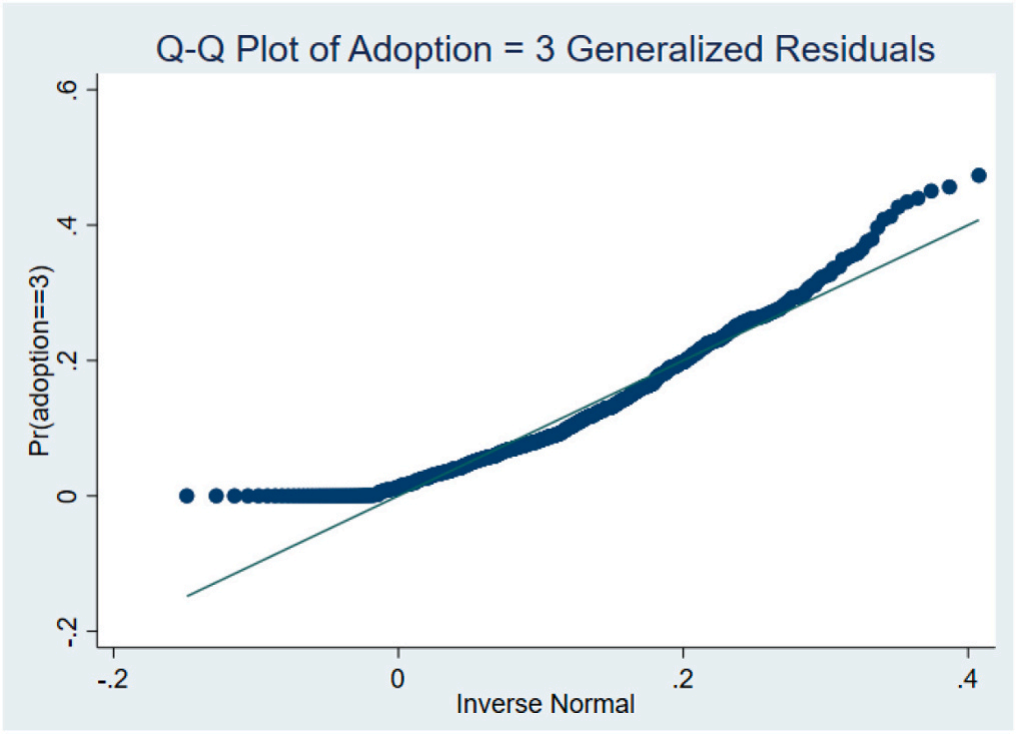


## Data Availability

The data that support the findings of this study are available from the corresponding author, NN, upon reasonable request.

## Appendix B Supplementary data

Supplementary material Image 3

## Footnotes

1 The predicted FSI values were obtained from the final adjusted OLS model using marginal effects estimation, holding market channel fixed (informal in Figure 1 and formal for Figure 2); error bars denote 95% CI)
