# Supplementary material for: Adoption of food safety measures in smallholder dairy farms in Kenya: Implications for milk safety and public health
Source: One Health. 2026 Jan 29;22:101342. doi: 10.1016/j.onehlt.2026.101342 (PMC12887420; doi:10.1016/j.onehlt.2026.101342)
Supplement: Supplementary file 1 — Supplementary material [file mmc1.pdf]

ILRI
INTERNATIONAL
LIVESTOCK RESEARCH
INSTITUTE

30 March 2017

Our Ref: ILRI-IREC2017-09

International Livestock Research Institute
P.O. Box 30709 00100
Nairobi, Kenya.

Dear Edgar Twine,

Re: Improving Milk Quality of Smallholder Dairy Systems in Kenya

Thank you for submitting your request for ethical approval to the ILRI Institutional Research Ethics Committee (ILRI IREC). ILRI IREC is accredited by the National Commission for Science, Technology and Innovation (NACOSTI) in Kenya.

This is to inform you that ILRI IREC has reviewed and approved your study titled **'Improving milk quality of smallholder dairy systems in Kenya'**. The approval period is March 30, 2017 to March 29, 2018 and is subject to compliance to the following requirements:

- Only approved documents will be used;
- All changes must be submitted for review and approval before implementation;
- Adverse events must be reported to ILRI IREC immediately;
- Submission of a request for renewal of approval at least 30 days prior to expiry of approval period; and
- Submission of an executive summary report within 90 days upon completion of the study.

Please do not hesitate to contact ILRI IREC on ILRIResearchcompliance@cgiar.org for any clarification or querry.

Yours Sincerely,

Dr. Silvia Alonso
Chair, ILRI Institutional Research Ethics Committee

Documents received & reviewed:

- Research Compliance Form and IREC Form 1
- Research Proposal
- Informed Consent Form
- Questionaire
- FGD questions
